# Genotype Impacts Axial Length Growth in Pseudophakic Eyes of Marfan Syndrome

**DOI:** 10.1167/iovs.64.10.28

**Published:** 2023-07-21

**Authors:** Ze-Xu Chen, Wan-Nan Jia, Tian-Hui Chen, Jia-Hao Hong, Yang Sun, Yan Liu, Ling-Hao Song, Yong-Xiang Jiang

**Affiliations:** 1 Eye Institute and Department of Ophthalmology, Eye and ENT Hospital, Fudan University, Shanghai, China; 2 NHC Key Laboratory of Myopia (Fudan University), Key Laboratory of Myopia, Chinese Academy of Medical Sciences, Shanghai, China; 3 Shanghai Key Laboratory of Visual Impairment and Restoration, Shanghai, China; 4 School of Computer Science, Fudan University Shanghai, China

**Keywords:** genotype-phenotype correlation, axial length (AL), intraocular lens (IOL), ectopia lentis (EL), Marfan syndrome (MFS)

## Abstract

**Purpose:**

The purpose of this study was to investigate the relationship between axial length (AL) growth and *FBN1* genotype in patients with Marfan syndrome (MFS) after lens surgery and customize the selection of intraocular lens (IOL) power.

**Methods:**

Patients with MFS who had lens surgery and primary IOL implantation received panel-based next-generation sequencing (NGS). The rate of axial length growth (RALG) was calculated using pre- and postoperative AL measurements and corrected log_10_-transformed age. A multivariable regression model of RALG was developed after analyzing the effect of *FBN1* genotypes and confounding factors.

**Results:**

A total of 139 probands of MFS with a median age at lens surgery of 6.25 years (interquartile range [IQR] = 4.67, 12.50 years) were followed up for a median duration of 2.08 years (IQR = 1.16, 3.00 years). The AL growth curve between the age of 3 and 15 years old was logarithmic. Dominant-negative (DN) variants affecting the disulfide-bridge forming cysteines and the conserved residues for calcium-binding had significantly higher RALG than DN variants affecting other structures (*P* = 0.001) but comparable to that of haplo-insufficiency variants (*P* = 1.000). Pre-operative AL (b = 0.563, *P* = 0.011) and genotype constant (b = 2.603, *P* = 0.011) were significantly associated with RALG in the final model. A Python-based calculator, Marfan IOL Calculator version 2.0, was programmed using the RALG to predict postoperative AL and customize IOL selection based on the ocular biometric parameters and *FBN1* genotype.

**Conclusions:**

*FBN1* genotype impacted the growth of AL in patients with MFS after IOL implantation. Knowing the *FBN1* genotype could help cataract surgeons to customize IOL selection.

Marfan syndrome (MFS; OMIM: 154700) is a multisystemic connective tissue disorder characterized by autosomal dominant inheritance and manifesting as ectopia lentis (EL), aortic aneurysm, and a series of skeletal deformities.^1^ In the latest nosology, MFS is mainly caused by *FBN1* variants, although the diagnosis is possible in the absence of identifiable variants.^2^ Despite the monogenic nature and high penetrance, one of the mysteries of MFS is the prominent individual variation in the severity of manifestations, the response to the therapies, and the progression of the disease.^3^ Recent years have seen the emergence of a number of genotype-phenotype correlations, providing illuminating insight that the clinical presentation and long-term prognosis could be partially explained by the *FBN1* genotypes,^4^^–^^6^ however, the related studies in the field of ophthalmology have significantly lagged behind.

Axial length (AL) refers to the distance from the anterior to posterior poles of the eyes on the axial plane and is longer in patients with MFS than in the general population.^7^ The growth of AL determines the myopic shift in pseudophakic eyes because of the fixed power of the intraocular lens (IOL) and the fully developed anterior eye after the age of 2 years.^8^ Cataract surgeons typically set undercorrection for pediatric patients to achieve emmetropia or mild to moderate myopia in adulthood when selecting IOL power for patients with EL. However, the amount of undercorrection depends on the surgeon's experience and the knowledge from the eyes of congenital cataract.^9^^–^^12^ Patients with slow AL growth suffered from refractory amblyopia due to excessive hyperopia, whereas those with rapid AL growth were troubled by dense myopia. In this study, we aimed to explore the clinical relevance between the AL growth rate and the *FBN1* genotype, providing novel insights into IOL power selection for patients with MFS.

## Methods

### Patient Eligibility and Ethics Statement

This retrospective study enrolled patients with MFS in the Eye and ENT Hospital, Fudan University, from 2015 to 2021. The inclusion criteria were: (1) patients diagnosed with MFS or potential MFS according to Ghent 2 nosology^2^; (2) probands carrying pathogenic or likely pathogenic *FBN1* variants; (3) patients receiving lens surgery with primary IOL implantation; and (4) eyes with both baseline AL before the surgery and the final AL measured after at least 1-year follow-up. Patients were excluded if they had: (1) an entirely dislocated lens into the anterior chamber or vitreous body; (2) a history of ocular trauma or surgery; and (3) pre-existing comorbidities or postoperative complications that affect the AL measurement or growth: IOL dislocation, glaucoma, macular hole, retinitis pigmentosa, and retinal detachment. This study was adherent to the Declaration of Helsinki and supervised by the Human Research Ethics Committee, Eye and ENT Hospital, Fudan University (ChiCTR2000039132) and informed written consent was obtained by all patients and their guardians for those under 18 years old.

### Ocular and General Examination

All enrolled patients underwent medical history recording and comprehensive ocular examinations. The patients received a slit-lamp examination under pupillary dilation. EL was diagnosed if the margin of the lens was visible or if there existed a tremor of the iris or lens. The severity of EL was categorized into mild, moderate, and severe according to our previous study.^13^ The same experienced optometrist measured the best-corrected visual acuity (BCVA) and spherical equivalent (SE). Ocular biometric parameters, including AL, corneal mean keratometry (Km), corneal astigmatism, anterior chamber depth (ACD), lens thickness (LT), and white-to-white measurement (WTW), and were obtained pre-operatively and in the follow-up visits by partial coherence interferometry (IOLMaster 500 and 700; Carl Zeiss Meditec AG, Jena, Germany). At least three valid readings were taken, and the mean value was used for analysis. For cardiovascular and skeletal evaluations, patients were referred to a general tertiary hospital, and the reports were documented.

### Genetic Screening and Mutation Classification

Panel-based next-generation sequencing (NGS) was carried out using DNA libraries from peripheral blood on an Illumina Novaseq 6000 platform (Illumina Inc., San Diego, CA, USA), as previously described.^14^ A tailored panel consisting of 41 genes for congenital EL was applied in cooperation with Amplicon Gene (Shanghai) from September 2021 onward (Supplementary Table S1). This EL-specific panel comprised genes that were either identified in our previous genetic screening of a Chinese cohort or associated with EL or Marfanoid habitus in previous studies.^15^^–^^17^ The reference sequence of the *FBN1* transcript was NM_000138. In silico analysis was performed using an integrated web tool, the Ensembl Variant Effect Predictor 105 (http://uswest.ensembl.org/info/docs/tools/vep/index.html), including splicing site prediction (SpliceAI), allele frequency annotation (gnomAD), and missense prediction (MutationTaster, PolyPhen, and SIFT). Candidate variants were verified by Sanger sequencing. Multiplex ligation‐dependent probe amplification (MLPA) was supplemented using SALSA MLPA Probemix kits (# P065‐C1/P066‐C1; MRC Holland) for patients with undetectable pathogenic variants after data re-analysis. The genotype-phenotype co-segregation analysis was performed on family members, and the pathogenicity of all variants was evaluated according to the American College of Medical Genetics and Genomics guidelines.^18^

*FBN1* variants were first generally categorized into two groups based on the mutation effects: the dominant-negative (DN) group, consisting of missense variants and in-frame deletions or insertions, and the haploinsufficiency (HI) group, including frameshift variants, nonsense variants, splicing variants, and intragenic deletions or duplications. Considering the inherent heterogeneity, DN variants were further classified based on two strategies (Supplementary Fig. S1A).^19^ The first strategy classified missense variants that affected critical structures, such as disulfide-bridge forming cysteines and the conserved motif for calcium-binding of the cb EGF-like domains, as DN (−Cys + CaB). The variants that did not affect the above residues were grouped as DN (others), which stood for the noncritical residues of FBN1 protein (Supplementary Fig. S1B). The second strategy categorized variants based on their location, including the FUN-EGF region (exons 1–11), neonatal region (exons 24–32), DN-CD region (exons 25–36 and exons 43–49), and TGFβ-regulating (TGFB) region (exons 44–49; Supplementary Fig. S1C).^19^

### Surgical Procedure

All the surgery was performed by the same surgeon (author Y.X.J.). The phacoemulsification and in-the-bag IOL implantation were planned, unless the continuous curvilinear capsulorhexis was unsuccessful. The phacoemulsification procedure and modified capsular tension ring (MCTR) implantation were conducted, as previously described.^20^ Due to the expiration of MCTR certification in mainland China since July 2020, the capsular tension ring and capsular hook (CTR-CH) implantation was performed instead.^21^ In brief, a preloaded CTR (ACPi-11; Bausch & Lomb, Rochester, NY, USA) was introduced into the capsular bag held by temporary capsule retractors. An implantable capsular hook was made by heating one end of a 5–0 polypropylene suture (Ethicon, Somerville, NJ, USA) using a cautery device. The hook was placed to engage the capsulorhexis rim by pulling the other end of the suture that penetrated through the sulcus about 1.5 mm posterior to the limbus. Two hooks were implanted if extensive zonular loss occurred. The suture was passed within the sclera and cut without any knot. A one-piece acrylic IOL was then injected into the capsular bag. In cases where a peripheral extension or tearing of the capsulorhexis rim occurred, a single-piece foldable IOL was sutured to the sclera using double-strand 9–0 polypropylene (MANI Inc., Tochigi, Utsunomiya, Japan) after anterior lensectomy combined with capsulotomy (23G; Alcon Laboratories Inc., Fort Worth, TX, USA) through a limbal approach.^22^

### Model Construction

We fit the AL model using the log_10_-transformed age according to previous studies.^23^^,^^24^ The age was corrected by adding 0.6 years to account for AL growth during embryonic eye formation.^25^ The AL measured before the surgery (preAL) and in the last follow-up (postAL) were used in the calculation of the rate of axial length growth (RALG):

$$\begin{array}{c} RALG=\frac{postAL\;-preAL}{log10\left(postAge+\;0.6\right)-log10\left(preAge+0.6\right)} \end{array}$$

The slope of the linear AL growth rate (kAL) was also calculated and compared with RALG:

$$\begin{array}{c} kAL=\frac{postAL\;-preAL}{postAge-preAge} \end{array}$$

We performed a multivariable linear regression analysis to predict RALG, dividing the patients into the test and validation groups using random sampling with a 4:1 allocation. The variables with a univariate *P* value < 0.05 were enrolled in the regression model using stepwise selection. The prediction error (PE), absolute error (AE), and mean absolute percentage error (MAPE) were calculated using the following formulas:

$$\begin{array}{c} PE=\;\frac{1}{N}\sum_{I=1}^{N}Ai-Pi \end{array}$$

$$\begin{array}{c} AE=\;\frac{1}{N}\sum_{I=1}^{N}\left|\left.Ai-Pi\right|\right. \end{array}$$

$$\begin{array}{c} MAPE=\;\frac{1}{N}\sum_{I=1}^{N}\left|\left.\frac{Ai-Pi}{Ai}\right|\right. \end{array}$$

Ai and Pi denote the actual and predicted AL at data point i and N refers to the total number.

To ensure easy implementation of the AL prediction model in clinical settings, we developed an IOL calculator based on the Haigis formula with Wang-Koch adjustment.^26^ This calculator calculated the IOL power using the postAL and target refraction at the desired age and then computed the immediate postoperative refraction by using the selected IOL power and the preAL. The parameters of three brands of IOL (Rayner C-flex Aspheric 920H or 970C, TECNIS PCB00 or ZCB00, and AcrySof IQ SN60AT or SN60WF) were integrated into the IOL calculator.

### Statistical Analysis

All statistical analyses were conducted in the SPSS 26.0 (IBM, Armonk, NY, USA). The normality of all the continuous data was analyzed by Kolmogorov–Smirnov test. Values were presented as the mean ± standard deviation (SD) that follow the Gaussian distribution. Otherwise, data were reported in the median and interquartile range (IQR). For two-group comparisons, the *t*-test or Mann–Whitney nonparametric test was applied to determine the difference. For multigroup comparisons, *P* values were derived from 1-way ANOVA or Kruskal-Wallis test (where appropriate) with post hoc Bonferroni adjustment. Categorical variables were summarized as the number of patients and the percentage, which were compared by χ2 analysis or Fisher's exact test. For all comparisons, the statistical significance was considered at the *P* < 0.05 (2-sided) level. The interocular correlation was analyzed by calculating the intraclass correlation coefficient (ICC).^27^^,^^28^

## Results

### Cohort Characteristics

**Figure 1.**
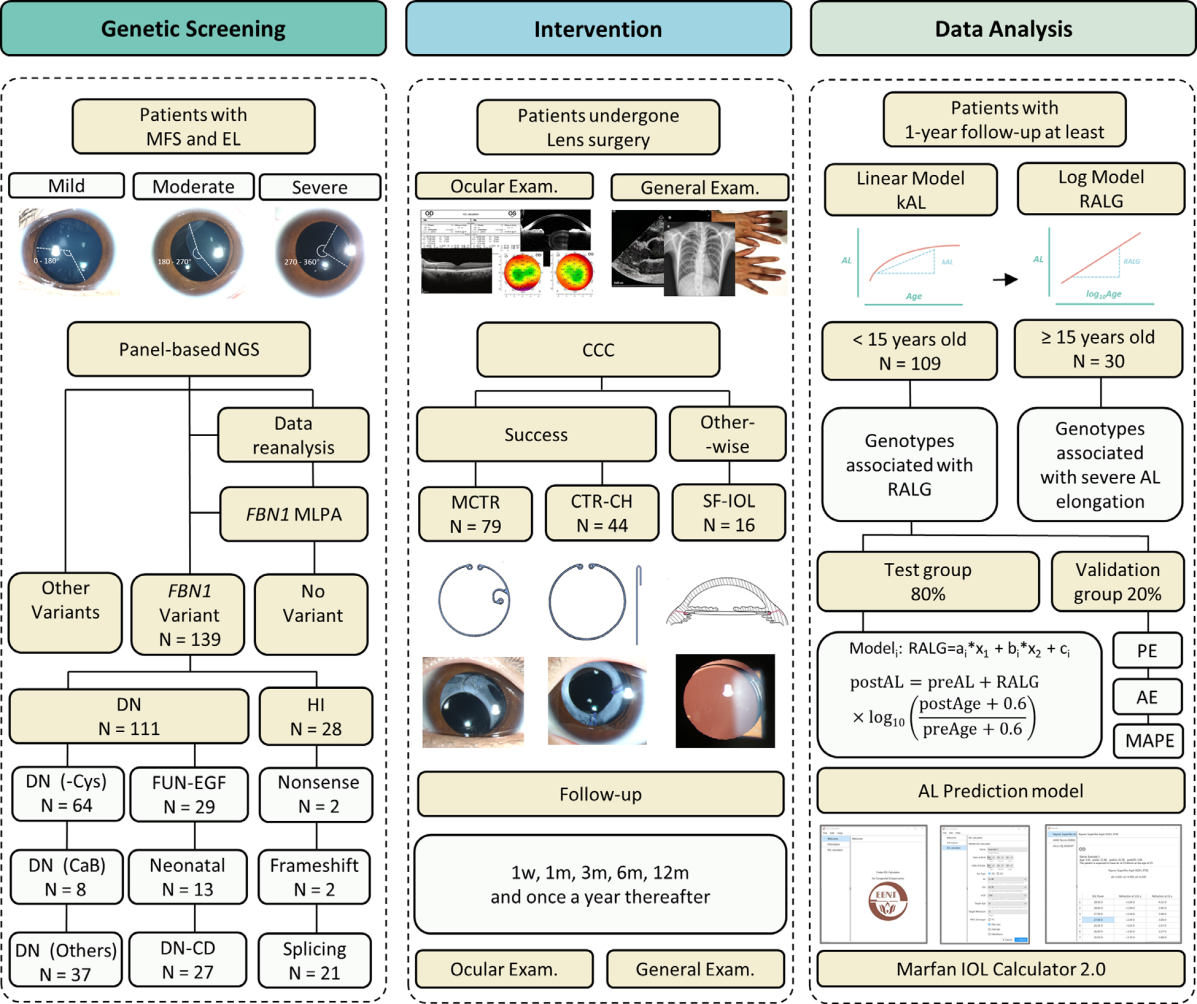
**The schematic diagram of the workflow.** The project consisted of three parts. First, patients with MFS and EL received panel-based NGS supplemented by data re-analysis and *FBN1* MLPA. The *FBN1* variants were further classified. Second, the patients had medical examinations, lens surgery, and follow-up visits. Last, the pre-operative and postoperative data were collected to build the AL prediction model based on the *FBN1* genotype. AL, axial length; AE, absolute error; CCC, continuous curvilinear capsulorhexis; CTR, capsular tension ring; CH, capsular hook; DN, dominant-negative; EL, ectopia lentis; HI, haplo-insufficiency; kAL, average AL growth per year in a linear model (mm/y); MAPE, mean absolute percentage error; MCTR, modified capsular tension ring; MFS, Marfan syndrome; MLPA, multiplex ligation-dependent probe amplification; NGS, next generation sequencing; PE, prediction error; RALG, rate of axial length growth in a logarithmic model (mm/log_10_y); SF-IOL, scleral-fixated intraocular lens.

A summary of the workflow is presented in Figure 1. A total of 139 eyes from 139 patients with MFS and EL were analyzed (Table 1). The median age at lens surgery was about 6 years old and about 80% of patients were under 15 years old. Patients were followed up for a median duration of 2.08 years. Missense variants of *FBN1* were the most prevalent among the participants, which were identified in about 80% of them (Supplementary Table S2). For patients who had bilateral surgery, only one eye was randomly selected for the remainder of the study. This approach was supported by the high correlation in RALG between the two eyes of the same patients with MFS (ICC = 0.790, *P* < 0.001).

**Table 1.** Characteristic of Patients With MFS and EL

Characteristics	Mean ± Standard Deviation (Range) or Median (IQR) or Number (%)^a^	
Eyes (right/left)	139 (71/68)	
M/F	86/53	
Bilateral/unilateral	128/11	
Age at surgery	6.25 (4.67, 12.50)	
	≤15 y old	109 (78.42%)
	> 15 y old	30 (21.58%)
Diagnosis	MFS	55 (39.57%)
	Potential MFS	84 (60.43%)
Ocular examination	SE/D	−8.13 (−13.75, −2.75)
	BCVA /LogMAR	0.52 (0.40, 0.82)
	AL/mm	23.67 (22.54, 25.58)
	Km/D	40.81 ± 2.83 (36.24, 45.26)
EL severity	Mild	39 (28.26%)
	Moderate	77 (55.80%)
	Severe	22 (15.94%)
Surgery	MCTR	79 (56.83%)
	CTR-CH	44 (31.65%)
	SF-IOL	16 (11.51%)
Follow-ups/y	2.08 (1.16, 3.00)	
	1 to 3 y	103 (74.10%)
	> 3 y	36 (25.90%)
IOL type	TECNIS ZCB00 or PCB00	56 (40.29%)
	AcrySof IQ SN60WF or SN60AT	52 (37.41%)
	Rayner C-flex Aspheric 970C or 970H	28 (20.14%)
	Other IOLs	3 (2.16%)
Genotype		
DN	Missense	109 (78.42%)
	In-frame insertion or deletion	2 (1.44%)
HI	Nonsense	2 (1.44%)
	Frameshift	2 (1.44%)
	Splicing mutation	21 (15.11%)
	Intragenic duplication or deletion	3 (2.16%)
ACMG criteria	Pathogenic	117 (84.17%)
	Likely pathogenic	22 (15.83%)
	Uncertain significance	0 (0.00%)

ACD, anterior chamber depth; ACMG, American College of Medical Genetics and genomics guidelines; AL, axial length; AST, corneal astigmatism; BCVA, best-corrected visual acuity; CH, capsular hook; CTR, capsular tension ring; D, diopter; DN, dominant negative effect; EL, ectopia lentis; HI, haploinsufficiency effect; IOL, intraocular lens; IQR, interquartile; Km, median keratometric of meridian; LogMAR, logarithm of the minimal angle of resolution; LT, lens thickness; MCTR, modified capsular tension ring; MFS, Marfan syndrome; SD, standard deviation; SF-IOL, scleral-fixated intraocular lens; WTW, white-to-white measurement.

a For continuous variables, normally distributed data are shown in the mean ± SD, while skewed data are shown in the median (interquartile range [IQR]).

### The Correlation Between AL Growth Rate and Genotype

**Figure 2.**
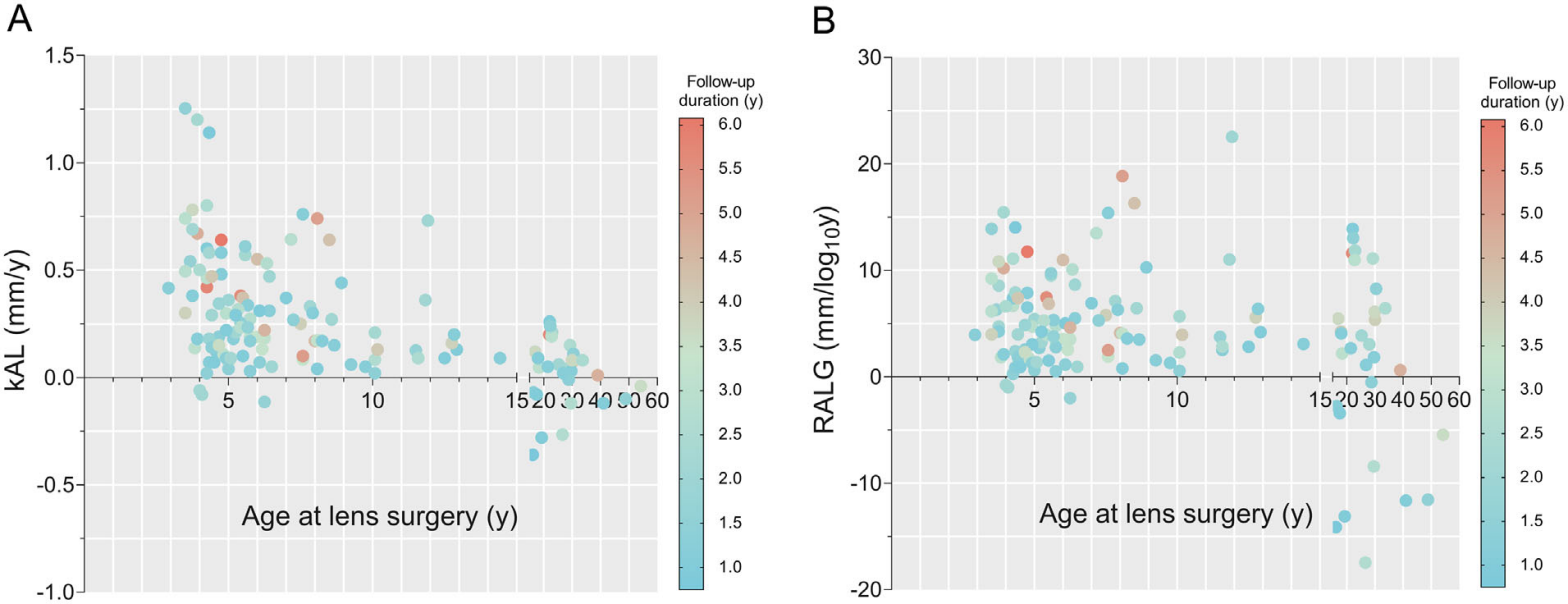
**The growth pattern of AL in patients with MFS and EL.** (**A**) The bubble chart of kAL by patients’ ages at lens surgery demonstrated the decreasing tendency of kAL toward older age. The follow-up duration was shown in gradient colors. (**B**) The bubble chart demonstrated that there was no correlation between RALG and age before 15 years old, and the distribution of RALG appeared almost symmetrical around the x-axis thereafter. EL, ectopia lentis; kAL, average AL growth per year in a linear model (mm/y); MFS, Marfan syndrome; RALG, rate of axial length growth in a logarithmic model (mm/log_10_y).

To investigate the correlation between genotype and phenotype in AL growth, we initially analyzed the average annual AL growth in both a linear model (kAL, mm/y) and a log-transformed model (RALG, mm/log_10_y; Fig. 2). The kAL decreased with the age at lens surgery, whereas the RALG did not correlate with age at lens surgery between 3 and 15 years old (b = 0.081, *P* = 0.616). Thus, the RALG was applied for the remaining of the study as it was not confounded by the age at lens surgery. Meanwhile, both kAL and RALG distributed almost symmetrically around the x-axis after 15 years old. Therefore, the patients were divided into 2 groups by the age of 15 years.

**Figure 3.**
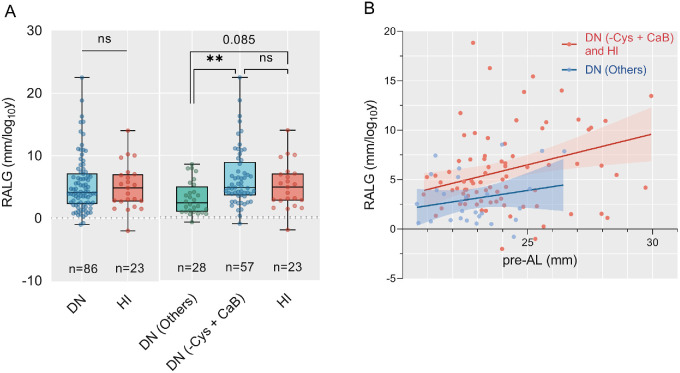
**Correlation of RALG with *FBN1* genotype and preoperative AL.** (**A**) Comparison of RALG across *FBN1* variants revealed higher RALG in the DN (−Cys + CaB) group than that in the DN (others) group. (**B**) Scatterplots of RALG and pre-operative AL showed a correlation of RALG with pre-operative AL between the patients with a DN (−Cys + CaB) or HI variant (b = 0.2233, 95% CI 0.1781 to 1.067, *P* = 0.007), which trended to be larger compared with those with a DN (others) variant (b = 0.3329, 95% CI –0.2997 to 1.069, *P* = 0.258). The shaded area demonstrates the confidence interval. DN (−Cys), DN variants eliminating the disulfide-bridge forming cysteines; DN (CaB), DN variants affecting the conserved calcium-binding motif; DN (others), DN variants affecting other residues; HI, haplo-insufficiency.

In 109 patients younger than 15 years old, the correlations between genotype and RALG were studied. Patients in the DN group and HI group showed similar RALG (Mann–Whitney test, *P* = 0.917). If the DN variants were subdivided, we found that patients with a DN (CaB) variant had higher RALG than those with a DN (others) variant (4.68 vs. 2.32, Kruskal-Wallis test, Bonferroni-adjusted, *P* = 0.013), whereas the difference between the RALG in patients with a DN (−Cys) and DN (CaB) variant was insignificant (5.016 vs. 4.675, Kruskal-Wallis test, Bonferroni-adjusted, *P* = 0.670). Thus, we combined the DN (−Cys) and DN (CaB) into a single category. We found that patients with a DN (−Cys + CaB) variant had significantly higher RALG than those harboring a DN (others) variant (4.80 vs. 2.32, Kruskal-Wallis test, *P* = 0.001). Patients in the HI group also had higher RALG than those in the DN (others) group, although the difference is not significant (4.86 vs. 2.32, Kruskal-Wallis test, *P* = 0.085; Fig. 3A). Considering the deleterious mutation effect of the DN (−Cys + CaB) and HI variants and the similar median RALG between the two groups, we combined the DN (−Cys + CaB) and HI variants as a single group for further analysis. As for the second classification scheme, DN variants in different regions did not correlate with RALG, including the FUN-EGF region, neonatal region, TGFB region, or DN-CD region (Supplementary Fig. S2). The potential confounding factors were also explored. RALG was independent of the age at surgery, sex, EL severity, unilateral or bilateral surgery, and pre-operative BCVA or SE in the univariate analysis (Supplementary Table S3). Patients with longer AL before the surgery had higher RALG after the lens surgery (*r* = 0.247, *P* = 0.010). A scatterplot showed the correlation of RALG with pre-operative AL between patients with different genotypes (Fig. 3B). Our results indicated that patients with a DN (−Cys + CaB) or HI variant tended to exhibit a higher RALG at varying pre-operative AL values when compared to those with a DN (others) variant. Other biometric parameters were not associated with RALG (see Supplementary Table S3). The surgery procedure (Kruskal-Wallis test, *P* = 0.115) and IOL brand (Kruskal-Wallis test, *P* = 0.812) did not influence the RALG either.

### Potential Risk Factors of Severe Continued AL Elongation

Continued AL growth was observed in subsets (8, 26.67%) of patients with MFS over 15 years old. Thus, we studied the risk factors of severe continued AL growth in 30 patients over 15 years old. To reduce measurement errors, the severe continued AL growth was defined as annual growth of 0.108 mm or greater in adulthood, according to a previous study.^29^ All the patients with severe AL elongation had either DN (−Cys + CaB) or HI variants, but not DN (others) variants, although the difference is not significant (Fisher's exact test, *P* = 0.073). There was no significant difference in the genotype distribution between patients who experienced severe continued AL growth and those who did not, among those over 15 years old (Supplementary Table S4). To compare demographic and biometric parameters between patients with nonsevere and severe continued AL elongation, we found that the two groups exhibited similar characteristics except for corneal astigmatism, which was smaller in the group with severe continued AL elongation (Supplementary Table S5).

### Multivariate Analysis of RALG

Multivariate linear regression analyses were performed to develop a prediction model of RALG in patients under the age of 15. After randomizing patients in a 4:1 ratio, 87 patients were enrolled in the test group and the remaining patients were used for external validation. The regression model included pre-operative AL and the aforementioned genotype groups. The final model incorporated pre-operative AL and the presence of DN (−Cys + CaB) and HI variants (Table 2). The resulting mathematical equation is as follows:

$$\begin{array}{ccc} & & postAL=preAL+RALG\times log10\left(\frac{postAge+0.6}{preAge+0.6}\right)\\ & & RALG\;=\;-10.231+\;0.563\;\times preAL+2.603\;\times c\\ & & DN\;\left(\mathrm{others}\right):c=0;\;DN\left(-Cys+CaB\right)\;or\;HI:c=1 \end{array}$$

**Table 2.** Multivariate Linear Regression Model of RALG

	RALG				
Parameter	*P* Value	Standardized β	Unstandardized B	95% CI of B	VIF
preAL/mm	0.011	0.268	0.563	0.132, 0.994	1.084
DN (−Cys + CaB) and HI	0.011	0.267	2.603	0.601, 4.604	1.084

preAL, pre-operative axial length; CI, confidence interval; DN (−Cys + CaB), dominant negative variants eliminating the disulfide-bond forming cysteines or affecting the conserved calcium-binding motif; HI, haploinsufficiency effect; RALG, rate of axial length growth; VIF, variance inflation factor.

Age was recorded in years and AL was recorded in millimeters. The genetic factor constant (c) was set to 1 for patients with DN (−Cys + CaB) or HI variants, and 0 for those with DN (other) variants.

### Model Validation

The PE, AE, and MAPE were evaluated in the validation group. The mean PE and AE of the postAL were −0.191 (95% confidence interval [CI] = −0.433 to 0.051) mm and 0.438 (95% CI = 0.238 to 0.589) mm, respectively. The MAPE was 1.603% (95% CI = 0.965%, 2.243%) for postAL.

### IOL Calculator Based on Predicted AL

**Figure 4.**
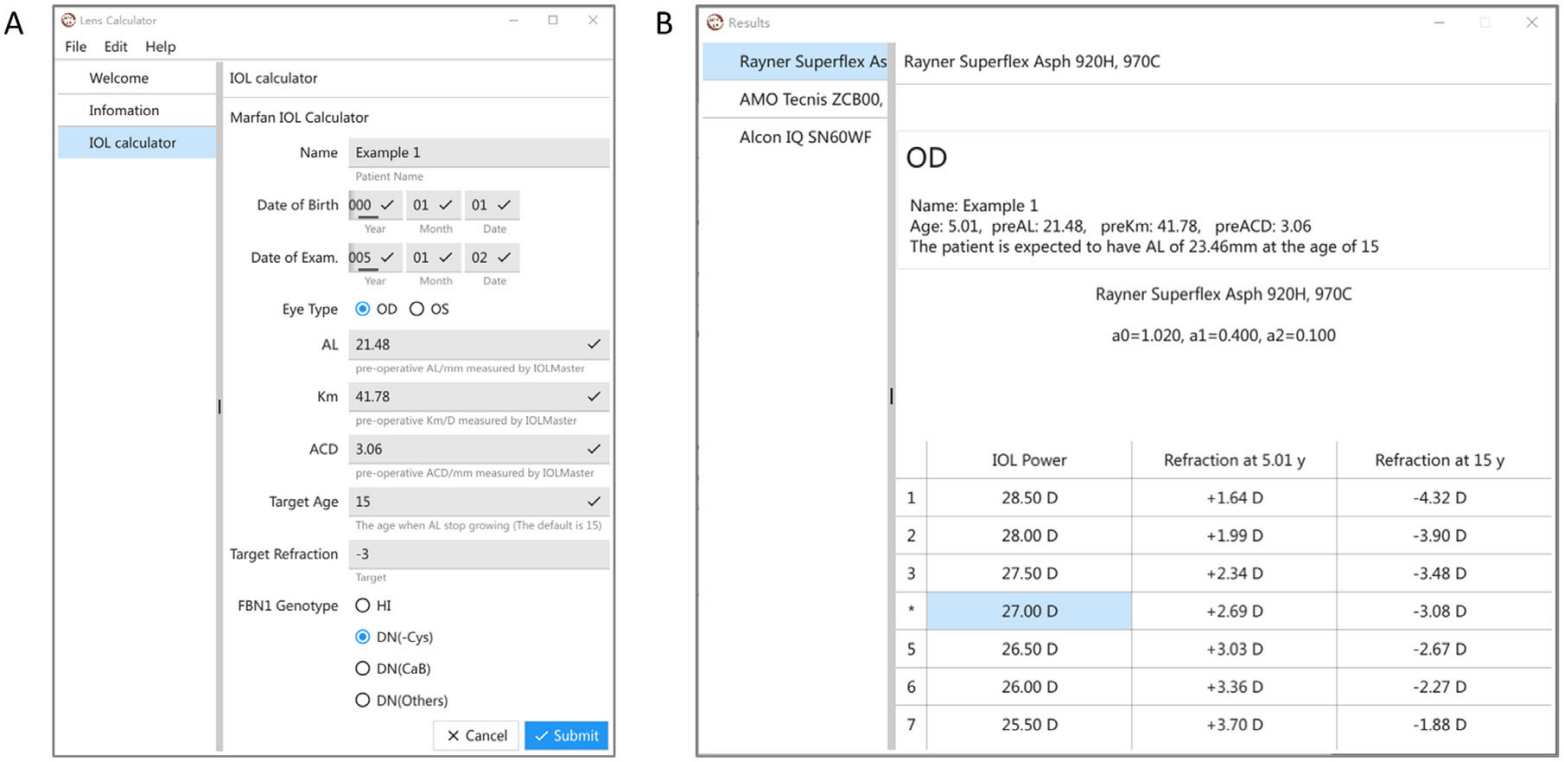
**The demonstration of Marfan IOL calculator 2.0.** (**A**) The entry screen included demographic information, ocular biometric parameters, the target refraction at the desired age (15 years old as default), and the *FBN1* genotype. (**B**) The export screen showed the predicted AL at the desired age, the recommended IOL power, the undercorrection after the surgery, and the future refraction at the targeted age. The constant values of three IOL models were built-in which can be chosen in the left column. ACD, anterior chamber depth; AL, axial length; IOL, intraocular lens; Km, median keratometry; DN, dominant-negative; DN (−Cys), DN variants eliminating the disulfide-bridge forming cysteines; DN (CaB), DN variants affecting the conserved calcium-binding motif; DN (others), DN variants affecting other residues; HI, haplo-insufficiency.

Knowing the future AL can help the surgeon to optimize IOL power selection in pediatric eyes. For example, consider a boy with MFS and a DN (others) variant who underwent lens surgery at 5 years old. The ocular biometrics of the surgical eye were as follows: AL = 21.48 mm; ACD = 3.06 mm; and Km = 41.78 diopters (D). If an IOL (Rayner, C-flex Aspheric 970H) of +28.00 D was implanted, his refraction would be +1.99 D after the surgery, according to the Haigis formula. Using the AL prediction model in this study, his RALG was predicted to be 1.862 mm/log_10_y, and he was expected to have a postAL of 22.31 mm, resulting in a refraction of −0.50 D at age 15 years. However, if another boy had the same biometric parameters but harbored a DN (−Cys) variant, he would have a RALG of 4.465 mm/log_10_y and a predicted AL of 23.46 mm at age 15 years. If the same IOL was implanted, he would have a refraction of −3.90 D at age 15 years. To facilitate the application of the AL prediction model, we developed an IOL power calculator, Marfan IOL Calculator 2.0 (Fig. 4, Supplementary Material S1). The calculator allowed input of the patient's date of birth, date of surgery, ocular biometric data, target refraction, and genotype, and displayed the IOL power and corresponding refraction data for both ages at the time of surgery and the desired age. In the case of the first patient, we recommended implanting an IOL of +31.50 D instead of +28.00 D if he desired better near vision when he grows up, resulting in a predicted refraction of −0.57 D after surgery and −3.26 D at age 15 years.

## Discussion

In the context of monogenic disorders, establishing a correlation between genotype and phenotype is crucial for risk assessment, prophylaxis, and clinical monitoring. With the growing knowledge of genotype-phenotype correlations in MFS, the identification of specific *FBN1* variants has been found to provide valuable information beyond the diagnosis. The ocular manifestations of MFS have been associated with certain *FBN1* variants, including EL severity, AL, central corneal thickness, and ciliary body cysts.^13^^,^^14^^,^^16^^,^^30^ However, whether the *FBN1* genotype can predict the progression of the ocular anomalies remains unknown. Through this study, we revealed the genotype-phenotype correlation between the RALG and *FBN1* genotypes, providing novel insights into predicting AL growth and setting undercorrection for young patients with MFS.

To date, a multitude of *FBN1* variants has been identified across the 65 coding exons and interspersed introns, encompassing the full spectrum of variant types.^31^ Most of the *FBN1* variants are unique to each pedigree.^32^ Therefore, the core concept when investigating the genotype-phenotype correlations in MFS is to make the appropriate classifications of the *FBN1* genotype. Early studies divided the *FBN1* variants into DN variants and HI variants based on the mutation effects. Patients with a DN variant were found to have a higher prevalence of EL, which has become widely accepted in this field of research.^19^ Some studies suggested a higher risk for aortic dilation in patients harboring an HI variant than those with DN variants.^33^^,^^34^ However, the correlation between HI variants and aortic dilation was not always consistent, even in some large cohorts.^3^^,^^35^ In our study, we found no significant difference in RALG between the DN and HI groups. Recent studies have recognized the heterogeneity of the DN variants and have further subgrouped based on two theories. One theory suggests that variants affecting the critical structures of FBN1, such as the paired cysteines forming disulfide-bridge and the conserved motif responsible for calcium-binding in the cb EGF-like domains, correlated with more severe MFS.^30^^,^^36^ Consistent with this theory, we found that patients with a DN (−Cys + CaB) variant had higher RALG and therefore faster myopic shift than those with a DN (others) variant. The other classification of DN variants is based on the location of the variants. We also compared RALG in patients harboring variants in some recognized regions, such as the neonatal region,^37^ the DN-CD region,^6^ and the TGFB region.^35^ The FUN-EGF region has been reported to be structurally significant in assembling *FBN1* monomers and thus was first applied in this study.^38^ However, no significant correlation was observed. The TGFB region was previously found to be associated with long pre-operative AL in our previous study.^13^ Although all the patients with DN variants in the TGFB region had high RALG, the difference was not significant, likely due to the limited number of patients enrolled. Because recent studies have suggested that DN (−Cys) variants and DN (CaB) variants were closer to HI variants in nature,^37^^,^^39^ we combined the DN (−Cys + CaB) and HI variants as a whole group in the multivariable regression model. We did not divide the HI variants into subgroups because previous studies have shown that the phenotypes of patients with a frameshift or nonsense variant are not associated with the termination site.^6^^,^^37^

The AL elongation in patients with MFS has been associated with myopia shift and increased risks for retinal detachment.^40^ However, not all patients with MFS exhibit long AL. Previous studies have shown that about 30% of patients with MFS had short AL in both childhood and adulthood.^41^^,^^42^ Our previous study had shown that certain variants in the *FBN1* gene, such as DN (−Cys) variants, DN (CaB) variants, and variants in the C terminus, were significantly associated with longer AL, indicating that the *FBN1* genotype may account for the AL difference among patients with MFS.^13^^,^^30^ However, longitudinal studies on AL growth in pseudophakic eyes of patients with MFS have been limited. Park et al. reported that patients with MFS have a higher average AL growth rate than the normal reference, and suggested more undercorrection to achieve emmetropia in later life.^43^ However, this conclusion was based on a small sample size of only 10 patients with MFS, and the authors did not specify the amount of undercorrection that should be reserved. Currently, most cataract surgeons use age-directed nomograms, similar to those used in congenital cataracts. These nomograms recommend setting the undercorrection at +3.0 D to +5.0 D at 3 to 4 years old, +1.0 D to +3.0 D at 5 to 6 years old, and −1.0 D to +1.0 D at 7 to 9 years old.^9^^–^^12^ However, the long-term refraction status is difficult to expect. Patients with both excessive hyperopia or dense myopia had compromised visual quality. Our study showed that the AL growth was significantly associated with *FBN1* genotype and pre-operative AL, but not with the surgical procedure or IOL brands used. Furthermore, the correlation between *FBN1* genotype and RALG was found to have clinical significance. In fact, our results indicated that patients with the same ocular biometrics at the age of 5 years but different *FBN1* genotypes could have a difference of approximately 3.0 D in their refraction when they reached adulthood, as demonstrated in the examples presented in the Results section. Because the prediction model included the pre-operative AL besides the *FBN1* genotype, we developed software that utilized our prediction model to calculate both immediate and future refraction for a given IOL power. We recommended setting the desired age at 15 years old, as we found that the average RALG decreased to zero after this age. Our study found that although a previous study suggested steady AL in adult patients with MFS,^44^ about 30% of adults in our study experienced severe continued AL growth, particularly those with a DN (−Cys + CaB) or HI variant. Although the difference did not reach statistical significance, this observation indicated that the patients harboring a DN (−Cys + CaB) or HI variants not only had a higher rate of AL growth but potentially had a higher risk of progressive myopic shift in adulthood than those with a DN (others) variant. Yet, this conclusion should be verified in a larger cohort through further studies.

This study presents a novel exploration of the relationship between the AL growth rate and the *FBN1* genotype. To improve the efficiency of genetic screening, we developed a new NGS panel, supplemented with *FBN1* MLPA, that not only increased sequencing depth but also reduced costs (data not shown). Despite these efforts, several limitations must be considered. First, not all the patients were followed up until adulthood, thus only the RALG was calculated and the future AL was predicted by a mathematical model. A longer follow-up duration with consecutive AL measurements would provide more robust evidence. Second, correlation analysis that did not reach statistical significance in certain genotype subgroups should be viewed with caution given our limited sample size. For example, some patients continued to experience AL growth beyond this age, but the risk factors were not clearly identified, likely due to the limited sample size. Additionally, our study only included patients who had undergone lens surgery, which may not be fully representative of the entire MFS population. Given the fact that EL severity did not correlate with RALG, we supposed that this selection bias might not affect the main conclusions. Finally, although our logarithmic model captured the rapid stage of AL growth in patients up to 2 years old, it remains unclear whether patients operated on before the age of 2 years can benefit from our model. Such patients were not included in this study, as they were more likely to be left aphakic and undergo secondary IOL implantation later in life. Whereas this study included 3- to 5-year-old patients, it is worth noting that placing MCTRs and sutured CTRs at this young age is not a routine practice in some countries, such as the United States.

## Conclusion

Our study demonstrated a significant correlation between AL growth and *FBN1* genotypes in 3- to 15-year-old patients with MFS following IOL implantation. Patients with DN (−Cys + CaB) or HI variants required more undercorrection than those with DN (others) variants. A multiple linear regression model was established to predict postoperative AL based on age, pre-operative AL, and *FBN1* genotype, allowing the surgeon to customize IOL power selection for individual patients with MFS.

## Supplementary Material

Supplement 1

Supplement 2

Supplement 3

Supplement 4

Supplement 5

Supplement 6

Supplement 7

Supplement 8
