# Supplementary material for: Genotype Impacts Axial Length Growth in Pseudophakic Eyes of Marfan Syndrome
Source: Invest Ophthalmol Vis Sci. 2023 Jul 21;64(10):28. doi: 10.1167/iovs.64.10.28 (PMC10365134; doi:10.1167/iovs.64.10.28)
Supplement: Supplement 1 [file iovs-64-10-28_s001.pdf]

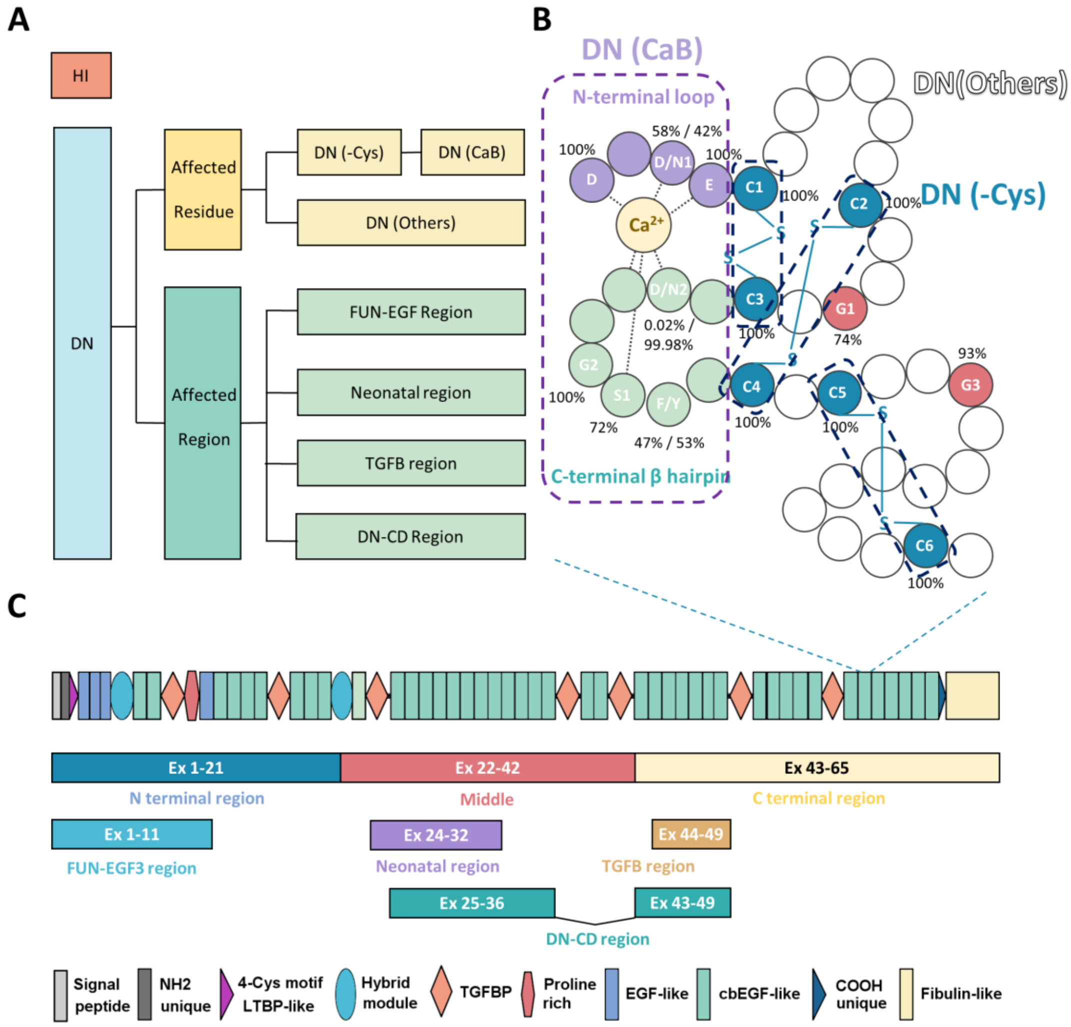

Supplementary Figure S1. The strategies for *FBN1* genotype classification.

(A). Variants were first broadly classified as HI and DN groups based on sequence information. DN variants were subdivided based on the affected residue or affected regions.

(B). The schematic diagram of cb EGF-like domain. The disulfide bridges (-S-S-) are annotated in cyan. The consensus sequence (D)X(D/N)(E/Q)Xm(D/N)Xn(F/Y), with m and n representing the variable number of residues, is responsible for calcium-binding, which consists of an N-terminal loop and a C-terminal β-hairpin. All the conservative amino acid residues were annotated with the percentage of identity. The DN (-Cys), DN (CaB), and DN (Others) variants were boxed out.

(C). The protein architecture of FBN1. The regions used in this study were marked in colored blocks, including N terminus (exons 1–21), middle region, (exons 22–42), C-terminus (exons 43–65), FUN-EGF region (exons 1–11), Neonatal region (exons 24–32), TGFB region (exons 44–49), and DN-CD region (exons 25–36 and exons 43–49). DN, dominant-negative; DN (-Cys), DN variants eliminating the disulfide-bridge forming cysteines; DN (CaB), DN variants affecting the conserved calcium-binding motif; DN (Others), DN variants affecting other residues; DN-CD, DN variants in tandem arrays of cb EGF-like domains; Ex, exon; FUN-EGF, fibrillin unique N-terminal (FUN) and the first three epidermal growth factor (EGF)-like domains; HI, haplo-insufficiency; TGFB, TGF-β regulating.
