# Supplementary material for: Genotype Impacts Axial Length Growth in Pseudophakic Eyes of Marfan Syndrome
Source: Invest Ophthalmol Vis Sci. 2023 Jul 21;64(10):28. doi: 10.1167/iovs.64.10.28 (PMC10365134; doi:10.1167/iovs.64.10.28)
Supplement: Supplement 2 [file iovs-64-10-28_s002.pdf]

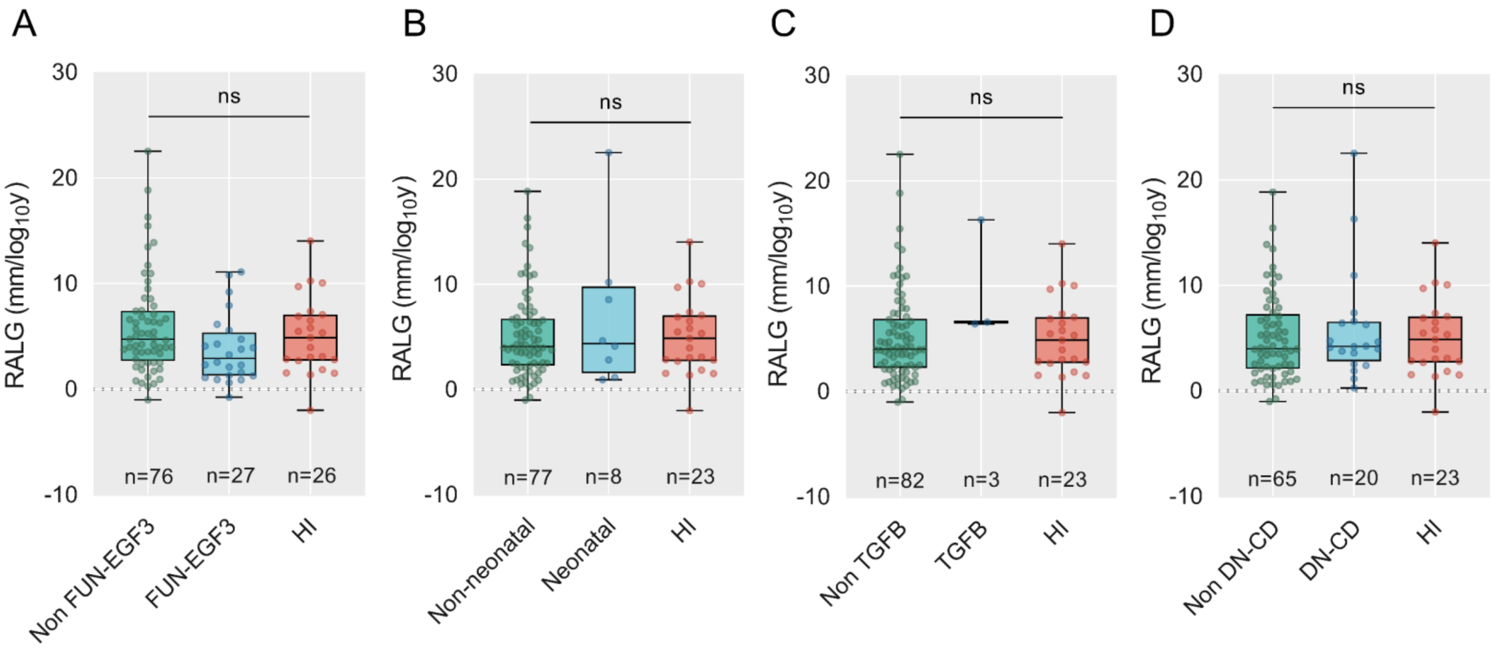

Supplementary Figure S2. Comparison of RALG across *FBN1* variants affecting different regions.

DN, dominant-negative; DN-CD, DN variants in tandem arrays of cb EGF-like domain; FUN-EGF, fibrillin unique N-terminal (FUN) and the first three epidermal growth factor (EGF)-like domains; HI, haplo-insufficiency; RALG, rate of axial length growth (mm/$\log_{10}$y); TGFB, TGF-β regulating.

^{ns} $P \geq 0.05$.
