# Supplementary material for: Genotype Impacts Axial Length Growth in Pseudophakic Eyes of Marfan Syndrome
Source: Invest Ophthalmol Vis Sci. 2023 Jul 21;64(10):28. doi: 10.1167/iovs.64.10.28 (PMC10365134; doi:10.1167/iovs.64.10.28)
Supplement: Supplement 3 [file iovs-64-10-28_s003.pdf]

Supplementary Material S1. The download link of Marfan IOL Calculator 2.0.

We provided the following links which contained the calculator used in this study:

For the international users:

https://drive.google.com/file/d/1LjHkT_vuN7vmtI2o7kzVuq67ok9qsuqm/view?usp=share_link

For the China mainland users:

https://pan.baidu.com/s/1vlUbLntrHWXAZ2Kd0nmXig passcode： 7fm1

If you have difficulties in getting and using the calculator, please contact Yongxiang Jiang via yongxiang_jiang@163.com
