# Supplementary material for: Genotype Impacts Axial Length Growth in Pseudophakic Eyes of Marfan Syndrome
Source: Invest Ophthalmol Vis Sci. 2023 Jul 21;64(10):28. doi: 10.1167/iovs.64.10.28 (PMC10365134; doi:10.1167/iovs.64.10.28)
Supplement: Supplement 4 [file iovs-64-10-28_s004.pdf]

Supplementary Table S1. Panel-based NGS of 41 genes tailored for MFS and congenital EL.

Code	Gene	GenBank transcript ID	Code	Gene	GenBank transcript ID
1	AASS	NM_005763	22	FOXE3	NM_012186
2	ACTA2	NM_001613	23	ITPR1	NM_002222
3	ADAMTS10	NM_030957	24	LTBP2	NM_000428
4	ADAMTS17	NM_139057	25	MOCS1	NM_005943
5	ADAMTSL4	NM_019032	26	MOCS2	NM_004531
6	ASPH	NM_004318	27	PAX6	NM_001604
7	B3GLCT	NM_194318	28	PITX2	NM_000325
8	CBS	NM_000071	29	PITX3	NM_005029
9	COL3A1	NM_000090	30	PXDN	NM_012293
10	COL4A1	NM_001303110	31	SLC16A12	NM_213606
11	COL4A5	NM_000495	32	SMAD2	NM_005901
12	COL5A1	NM_000093	33	SMAD3	NM_005902
13	COL5A2	NM_000393	34	SMAD4	NM_005359
14	COL18A1	NM_001379500	35	SUOX	NM_000456
15	CPAMD8	NM_015692	36	TGFB2	NM_003238
16	CRYGS	NM_017541	37	TGFB3	NM_003239
17	CYP1B1	NM_000104	38	TGFBR1	NM_001130916
18	FBN1	NM_000138	39	TGFBR2	NM_001024847
19	FBN2	NM_001999	40	VSX2	NM_182894
20	FLNA	NM_001110556	41	WDR8	NM_017818
21	FOXC1	NM_001453			

EL, ectopia lentis; MFS, Marfan syndrome; NGS, next generation sequencing;
