# Supplementary material for: Genotype Impacts Axial Length Growth in Pseudophakic Eyes of Marfan Syndrome
Source: Invest Ophthalmol Vis Sci. 2023 Jul 21;64(10):28. doi: 10.1167/iovs.64.10.28 (PMC10365134; doi:10.1167/iovs.64.10.28)
Supplement: Supplement 5 [file iovs-64-10-28_s005.pdf]

Supplementary Table S2. ***FBN1*** **mutations in this cohort of patients.**

Proband ID	Age at surgery	Gender	Status	Variant		Mutation effect		Mutation region		ACMG
				Nucleotide	Amino acid	Type	Group	DNA	Protein	
EENT001	30.00	F	Het	c.2920C>T	p.R974C	Missense	DN(Other)	Exon24	TGFBP #03	LP
EENT002	8.00	F	Het	c.3794G>A	p.C1265Y	Missense	DN(-Cys)	Exon30	cb EGF-like #16	P
EENT003	4.25	M	Het	c.5788+5G>A	-	Splicing	HI	Intron47	-	P
EENT004	21.92	F	Het	c.2414G>A	p.C805Y	Missense	DN(-Cys)	Exon19	cb EGF-like #08	P
EENT005	8.50	F	Het	c.5801G>C	p.C1934S	Missense	DN(-Cys)	Exon47	cb EGF-like #29	P
EENT006	7.58	M	Het	c.4096G>A	p.E1366K	Missense	DN(CaB)	Exon33	cb EGF-like #19	P
EENT007	5.75	M	Het	c.2848T>C	p.C950R	Missense	DN(-Cys)	Exon23	cb EGF-like #10	P
EENT008	3.83	M	Het	c.1879C>T	p.R627C	Missense	DN(Other)	Exon15	cb EGF-like #06	LP
EENT009	10.08	F	Het	c.1709G>C	p.C570S	Missense	DN(-Cys)	Exon13	cb EGF-like #04	P
EENT010	4.25	M	Het	c.2804G>A	p.C935Y	Missense	DN(-Cys)	Exon23	cb EGF-like #10	P
EENT011	10.08	F	Het	c.479G>A	p.C160Y	Missense	DN(-Cys)	Exon5	EGF-like #03	LP
EENT012	11.83	M	Het	c.1884C>G	p.C628W	Missense	DN(-Cys)	Exon15	cb EGF-like #06	LP
EENT013	9.75	F	Het	c.184C>T	p.R62C	Missense	DN(Other)	Exon2	4-Cys motif LTBP-like	LP
EENT014	8.08	M	Het	c.1948C>T	p.R650C	Missense	DN(Other)	Exon15	cb EGF-like #06	LP
EENT015	8.92	M	Het	c.1693C>T	p.R565X	Nonsense	HI	Exon13	-	P
EENT016	12.92	F	Het	c.4285T>C	p.C1429R	Missense	DN(-Cys)	Exon34	cb EGF-like #20	LP
EENT017	5.00	M	Het	c.1727G>A	p.C576Y	Missense	DN(-Cys)	Exon14	cb EGF-like #05	P
EENT018	6.17	M	Het	c.4537T>C	p.C1513R	Missense	DN(-Cys)	Exon36	cb EGF-like #22	P
EENT019	6.42	F	Het	c.1638C>G	p.C546W	Missense	DN(-Cys)	Exon13	cb EGF-like #04	P
EENT020	5.75	M	Het	c.4982G>A	p.G1661E	Missense	DN(Other)	Exon40	cb EGF-like #24	LP
EENT021	6.25	M	Het	c.6697C>G	p.P2233A	Missense	DN(Other)	Exon54	cb EGF-like #34	LP

EENT022	5.75	M	Het	c.2724T>G	p.C908W	Missense	DN(-Cys)	Exon22	Hybrid motif #02	LP
EENT023	4.25	F	Het	c.6379+1del	-	Splicing	HI	Intron52	-	P
EENT024	29.25	M	Het	c.1600T>C	p.C534R	Missense	DN(-Cys)	Exon13	cb EGF-like #04	P
EENT025	28.00	F	Het	c.6496+1G>A	-	Splicing	HI	intorn53	-	P
EENT026	29.58	F	Het	c.6688T>C	p.C2230R	Missense	DN(-Cys)	Exon54	cb EGF-like #34	P
EENT027	2.92	M	Het	c.2722T>C	p.C908R	Missense	DN(-Cys)	Exon22	Hybrid motif #02	P
EENT028	7.83	M	Het	c.4601G>T	p.C1534F	Missense	DN(-Cys)	Exon37	TGFBP #04	P
EENT029	6.25	M	Het	c.6998-2A>G	-	Splicing	HI	Intron57	-	P
EENT030	5.42	F	Het	c.4694C>T	p.S1565F	Missense	DN(Other)	Exon37	TGFBP #04	P
EENT031	5.17	M	Het	c.4930C>T	p.R1644X	Nonsense	HI	Exon39	-	P
EENT032	5.58	F	Het	c.1904A>G	p.Y635C	Missense	DN(CaB)	Exon15	cb EGF-like #06	P
EENT033	16.33	M	Het	c.199T>C	p.C67R	Missense	DN(-Cys)	Exon2	4-Cys motif LTBP-like	P
EENT034	4.75	F	Het	c.2728+2T>C	-	Splicing	HI	Intron22	-	P
EENT035	11.92	M	Het	c.3476G>T	p.C1159F	Missense	DN(-Cys)	Exon28	cb EGF-like #14	P
EENT036	8.67	M	Het	c.2054G>A	p.C685Y	Missense	DN(-Cys)	Exon16	TGFBP #02	P
EENT037	8.58	M	Het	c.6158G>T	p.C2053F	Missense	DN(-Cys)	Exon49	cb EGF-like #31	P
EENT038	5.42	M	Het	c.718C>T	p.R240C	Missense	DN(Other)	Exon6	Hybrid module #01	P
EENT039	25.25	M	Het	c.2168-1G>C	-	Splicing	HI	Intron18	-	P
EENT040	7.58	M	Het	c.4143_4145del	p.K1381del	Inframe deletion or insertion	DN(Inframe)	Exon33	cb EGF-like #19	LP
EENT041	7.00	F	Het	c.6379G>T		Splicing	HI	Exon51	-	P
EENT042	4.58	M	Het	c.640G>a	p.G214S	Missense	DN(Other)	Exon6	Hybrid motif #01	P

EENT043	6.42	M	Het	allel deletion		Allel deletion	HI	-	-	P
EENT044	3.67	M	Het	c.4120T>C	p.C1374R	Missense	DN(-Cys)	Exon33	cb EGF-like #19	P
EENT045	3.92	F	Het	c.2369G>A	p.C790Y	Missense	DN(-Cys)	Exon19	cb EGF-like #08	P
EENT046	6.08	M	Het	c.2741G>T	p.C914F	Missense	DN(-Cys)	Exon23	cb EGF-like #10	P
EENT047	4.00	F	Het	c.5800T>C	p.C1934R	Missense	DN(-Cys)	Exon47	cb EGF-like #29	P
EENT048	4.67	F	Het	c.184C>T	p.R62C	Missense	DN(Other)	Exon2	4-Cys motif LTBP-like	P
EENT049	4.00	M	Het	c.641G>A	p.G214D	Missense	DN(Other)	Exon6	Hybrid module #01	P
EENT050	6.00	F	Het	c.1633C>T	p.R545C	Missense	DN(Other)	Exon13	cb EGF-like #04	P
EENT051	19.25	M	Het	c.290C>G	p.P97R	Missense	DN(Other)	Exon3	EGF-like #01	LP
EENT052	6.50	M	Het	c.2860C>T	p.R954C	Missense	DN(Other)	Exon24	TGFBP #03	P
EENT053	4.33	M	Het	c.5788+5G>A	-	Splicing	HI	Intron47	-	P
EENT054	3.50	F	Het	c.640G>A	p.G214S	Missense	DN(Other)	Exon6	Hybrid module #01	P
EENT055	5.25	M	Het	c.718C>T	p.R240C	Missense	DN(Other)	Exon6	Hybrid module #01	P
EENT056	8.08	F	Het	c.1868G>T	p.C623F	Missense	DN(-Cys)	Exon15	cb EGF-like #06	P
EENT057	3.92	M	Het	c.2872T>A	p.C958S	Missense	DN(-Cys)	Exon24	TGFBP #03	LP
EENT058	4.33	M	Het	c.184C>T	p.R62C	Missense	DN(Other)	Exon2	4-Cys motif LTBP-like	P
EENT059	4.67	F	Het	c.184C>T	p.R62C	Missense	DN(Other)	Exon2	4-Cys motif LTBP-like	P
EENT060	4.33	F	Het	c.4454G>A	p.C1485Y	Missense	DN(-Cys)	Exon35	cb EGF-like #21	P

EENT061	10.08	F	Het	c.1633C>T	p.R545C	Missense	DN(Other)	Exon13	cb EGF-like #04	P
EENT062	4.92	F	Het	c.4096G>A	p.E1366K	Missense	DN(CaB)	Exon33	cb EGF-like #19	P
EENT063	30.42	F	Het	c.5076_5078del	p.R1692del	Inframe deletion or insertion	DN(Inframe)	Exon41	TGFBP #05	P
EENT064	3.75	F	Het	c.355T>C	p.C119R	Missense	DN(-Cys)	Exon4	EGF-like #02	LP
EENT065	6.08	M	Het	c.3997T>C	p.C1333R	Missense	DN(-Cys)	Exon32	cb EGF-like #18	P
EENT066	5.58	F	Het	c.5545+1G>C	-	Splicing	HI	Intron45	-	P
EENT067	29.67	M	Het	c.2861G>C	p.R954P	Missense	DN(Other)	Exon24	TGFBP #03	P
EENT068	48.83	F	Het	c.3244G>T	p.G1082C	Missense	DN(Other)	Exon26	cb EGF-like #12	LP
EENT069	3.75	F	Het	c.1380T>G	p.C460W	Missense	DN(-Cys)	Exon11	EGF-like #04	LP
EENT070	4.42	F	Het	c.2810G>A	p.C937Y	Missense	DN(-Cys)	Exon23	cb EGF-like #10	P
EENT071	4.67	F	Het	c.5788+5G>A	-	Splicing	HI	Intron47	-	P
EENT072	4.25	M	Het	c.407G>T	p.C136F	Missense	DN(-Cys)	Exon4	EGF-like #02	LP
EENT073	39.00	M	Het	c.6322C>T	p.R2108C	Missense	DN(Other)	Exon51	TGFBP #06	P
EENT074	5.75	M	Het	c.8264delG	p.S2755Ifs*23	Frameshift	HI	Exon65	-	P
EENT075	7.50	M	Het	c.4817-42_4817-7del	-	Splicing	HI	Intron39	-	P
EENT076	10.17	F	Het	c.2432G>A	p.C811Y	Missense	DN(-Cys)	Exon20	cb EGF-like #09	P
EENT077	6.00	M	Het	c.4096G>A	p.E1366K	Missense	DN(CaB)	Exon33	cb EGF-like #19	P
EENT078	5.42	M	Het	c.4816+1G>A	-	Splicing	HI	Intron39	-	P
EENT079	4.08	M	Het	c.1670G>A	p.C557Y	Missense	DN(-Cys)	Exon13	cb EGF-like #04	P
EENT080	3.75	M	Het	c.305G>T	p.C102F	Missense	DN(-Cys)	Exon3	EGF-like #01	P
EENT081	5.50	M	Het	c.2687G>A	p.C896Y	Missense	DN(-Cys)	Exon22	Hybrid motif #02	P
EENT082	12.75	M	Het	c.364C>T	p.R122C	Missense	DN(Other)	Exon4	EGF-like #02	P

EENT083	17.92	M	Het	c.5840G>A	p.C1947Y	Missense	DN(-Cys)	Exon47	cb EGF-like #29	P
EENT084	16.92	M	Het	c.6354C>T	-	Splicing	HI	Exon51	-	P
EENT085	29.92	M	Het	c.1426T>C	p.C476R	Missense	DN(-Cys)	Exon11	EGF-like #04	P
EENT086	54.17	F	Het	c.3244G>T	p.G1082C	Missense	DN(Other)	Exon26	cb EGF-like #12	LP
EENT087	4.83	M	Het	c.1948C>T	p.R650C	Missense	DN(Other)	Exon15	cb EGF-like #06	P
EENT088	33.67	F	Het	c.5885_5895del	p.Y1962Sfs*11	Frameshift	HI	Exon47	-	P
EENT089	3.75	M	Het	c.3037G>A	P.G1013R	Missense	DN(Other)	Exon24	TGFBP #03	P
EENT090	5.67	M	Het	Exon 34 duplication	-	Intragenic dupilication	HI	-	-	P
EENT091	41.00	F	Het	c.3725G>A	p.C1242Y	Missense	DN(-Cys)	Exon30	cb EGF-like #16	P
EENT092	5.67	M	Het	c.1463G>A	p.C488Y	Missense	DN(-Cys)	Exon11	EGF-like #04	LP
EENT093	18.33	F	Het	c.5782T>C	p.C1928R	Missense	DN(-Cys)	Exon46	cb EGF-like #28	P
EENT094	5.00	F	Het	c.364C>T	p.R122C	Missense	DN(Other)	Exon4	EGF-like #02	P
EENT095	11.50	M	Het	c.4538G>C	p.C1513S	Missense	DN(-Cys)	Exon36	cb EGF-like #22	P
EENT096	22.92	F	Het	c.4222T>G	p.C1408G	Missense	DN(-Cys)	Exon34	cb EGF-like #20	P
EENT097	3.50	M	Het	c.2432G>A	p.C811Y	Missense	DN(-Cys)	Exon20	cb EGF-like #09	P
EENT098	5.00	F	Het	c.188A>G	p.Y63C	Missense	DN(Other)	Exon2	4-Cys motif LTBP-like	P
EENT099	9.25	M	Het	c.5788+2T>G	-	Splicing	HI	Intron47	-	P
EENT100	4.42	F	Het	c.5918-2A>G	-	Splicing	HI	Intron48	-	P
EENT101	28.75	M	Het	c.4588C>T	p.R1530C	Missense	DN(Other)	Exon37	TGFBP #04	P
EENT102	22.75	F	Het	c.5992T>G	p.C1998G	Missense	DN(-Cys)	Exon48	cb EGF-like #30	P
EENT103	3.50	M	Het	c.4217A>G	p.D1406G	Missense	DN(CaB)	Exon34	cb EGF-like #20	P
EENT104	4.92	M	Het	c.266G>C	p.C89S	Missense	DN(-Cys)	Exon3	EGF-like #01	P
EENT105	22.08	M	Het	c.1759T>C	p.C587R	Missense	DN(-Cys)	Exon14	cb EGF-like #05	P

EENT106	7.92	F	Het	c.1511G>A	p.C504Y	Missense	DN(-Cys)	Exon12	cb EGF-like #03	P
EENT107	5.33	F	Het	c.4981G>C	p.G1661R	Missense	DN(Other)	Exon40	cb EGF-like #24	P
EENT108	4.75	M	Het	c.1948C>T	p.R650C	Missense	DN(Other)	Exon15	cb EGF-like #06	P
EENT109	11.58	F	Het	c.2369G>C	p.C790S	Missense	DN(-Cys)	Exon19	cb EGF-like #08	P
EENT110	16.08	M	Het	c.6772T>C	p.C2258R	Missense	DN(-Cys)	Exon55	cb EGF-like #35	P
EENT111	22.25	M	Het	c.2201G>T	p.C734F	Missense	DN(-Cys)	Exon18	cb EGF-like #07	P
EENT112	5.08	M	Het	c.5066A>T	-	Splicing	HI	Exon41	-	P
EENT113	6.25	M	Het	c.4022A>G	p.N1341S	Missense	DN(CaB)	Exon32	cb EGF-like #18	P
EENT114	12.50	M	Het	c.2860C>T	p.R954C	Missense	DN(Other)	Exon24	TGFBP #03	P
EENT115	8.08	M	Het	c.364C>T	p.R122C	Missense	DN(Other)	Exon4	EGF-like #02	P
EENT116	14.42	F	Het	c.2419+1G>A	-	Splicing	HI	Intron20	-	P
EENT117	3.50	M	Het	c.239G>A	p.C80Y	Missense	DN(-Cys)	Exon2	4-Cys motif LTBP-like	P
EENT118	5.25	M	Het	c.4283G>C	p.R1428P	Missense	DN(CaB)	Exon34	cb EGF-like #20	LP
EENT119	4.42	M	Het	c.4260C>G	p.C1420W	Missense	DN(-Cys)	Exon34	cb EGF-like #20	P
EENT120	21.42	M	Het	c.718C>T	p.R240C	Missense	DN(Other)	Exon6	Hybrid module #01	P
EENT121	6.33	M	Het	Exon 39-42 deletion		Intragenic deletion	HI	-	-	P
EENT122	4.25	M	Het	c.4205G>A	p.C1402Y	Missense	DN(-Cys)	Exon33	cb EGF-like #19	P
EENT123	18.00	M	Het	c.176G>A	p.C59Y	Missense	DN(-Cys)	Exon2	4-Cys motif LTBP-like	P
EENT124	17.50	M	Het	c.3463G>A	-	Splicing	HI	Exon27	-	P
EENT125	4.33	F	Het	c.364C>T	p.R122C	Missense	DN(Other)	Exon4	EGF-like #02	P
EENT126	12.83	M	Het	c.2762G>A	p.C921Y	Missense	DN(-Cys)	Exon23	cb EGF-like #10	P

EENT127	26.83	M	Het	c.2495G>C	p.C832S	Missense	DN(-Cys)	Exon20	cb EGF-like #09	P
EENT128	8.25	M	Het	c.5417G>A	p.C1806Y	Missense	DN(-Cys)	Exon43	cb EGF-like #25	P
EENT129	4.92	F	Het	c.538+1G>A	-	Splicing	HI	Intron6	-	P
EENT130	5.50	F	Het	c.6740A>G	-	Splicing	HI	Exon55	-	P
EENT131	4.50	M	Het	c.718C>T	p.R240C	Missense	DN(Other)	Exon6	Hybrid module #01	P
EENT132	11.58	F	Het	c.4460-8G>A	-	Splicing	HI	Intron36	-	P
EENT133	7.25	M	Het	c.2375G>T	p.C792F	Missense	DN(-Cys)	Exon19	cb EGF-like #08	P
EENT134	26.58	M	Het	c.1879C>T	p.R627C	Missense	DN(Other)	Exon15	cb EGF-like #06	LP
EENT135	4.67	M	Het	c.386G>T	p.C129F	Missense	DN(-Cys)	Exon4	EGF-like #02	P
EENT136	4.75	M	Het	c.2180G>A	p.C727Y	Missense	DN(-Cys)	Exon18	cb EGF-like #07	P
EENT137	3.92	F	Het	c.6251G>C	p.C2084S	Missense	DN(-Cys)	Exon50	TGFBP #06	LP
EENT138	7.17	M	Het	c.6388G>A	p.E2130K	Missense	DN(CaB)	Exon52	cb EGF-like #32	P
EENT139	7.58	F	Het	c.1760G>T	p.C587F	Missense	DN(-Cys)	Exon14	cb EGF-like #05	P

ACMG, American College of Medical Genetics and genomics guidelines; DN, dominant negative effect; DN (-Cys), DN variants eliminating the disulfide-bond forming cysteines; DN (CaB), DN variants affecting the conserved calcium-binding motif; DN (Others), DN variants affecting other residues; DN (Inframe), inframe insertions or deletions; F, female; Het, heterozygous; HI, haploinsufficiency effect; LP, likely pathogenic; M, male; P, pathogenic;
