# Supplementary material for: Genotype Impacts Axial Length Growth in Pseudophakic Eyes of Marfan Syndrome
Source: Invest Ophthalmol Vis Sci. 2023 Jul 21;64(10):28. doi: 10.1167/iovs.64.10.28 (PMC10365134; doi:10.1167/iovs.64.10.28)
Supplement: Supplement 6 [file iovs-64-10-28_s006.pdf]

Supplementary Table S3. The univariate analysis of the influencing factors of RALG other than genotype.

Variable	RALG		
	n	r	*P* value
Age at surgery/y	109	-0.035	0.719
Sex	109	0.054	0.580
EL severity	108	0.073	0.454
Laterality	109	-0.022	0.817
BCVA/LogMAR	101	0.088	0.380
SE/D	91	0.036	0.733
preAL/mm	109	0.247	0.010
preKm/D	109	-0.067	0.490
preAST/D	109	0.028	0.772
preACD/D	86	0.099	0.364
preLT/mm	52	-0.232	0.098
preWTW/mm	76	-0.026	0.824

ACD, anterior chamber depth; AL, axial length; AST, corneal astigmatism; BCVA, best-corrected visual acuity; CI, confidence interval; D, diopter; EL, ectopia lentis; Km, median keratometry of meridian; LogMAR, logarithm of the minimal angle of resolution; LT, lens thickness; RALG, rate of axial length growth; SD, standard deviation; SE, spherical equivalent; WTW, white-to-white measurement.
