# Supplementary material for: Genotype Impacts Axial Length Growth in Pseudophakic Eyes of Marfan Syndrome
Source: Invest Ophthalmol Vis Sci. 2023 Jul 21;64(10):28. doi: 10.1167/iovs.64.10.28 (PMC10365134; doi:10.1167/iovs.64.10.28)
Supplement: Supplement 7 [file iovs-64-10-28_s007.pdf]

Supplementary Table S4. Relationship between *FBN1* genotype and the prevalence of severe continued AL elongation in MFS patients over 15 years old.

Genotype#	Continued AL growth after the age of 15			
	Total n	Severe n (%)	Non-severe n (%)	*P* value
DN	25	7 (28.0%)	18 (72.0%)	1.000
HI	5	1 (20.0%)	4 (80.0%)	
DN (Others)	9	0 (0.0%)	9 (100.0%)	0.073
DN (-Cys + CaB)	15	6 (40.0%)	9 (60.0%)	
HI	5	1 (20.0%)	4 (80.0%)	
Non DN-CD	17	4 (23.5%)	13 (76.5%)	1.000
DN-CD	7	2 (28.6%)	5 (71.4%)	
HI	5	1 (20.0%)	4 (80.0%)	
Non FUN	19	6 (31.6%)	13 (68.4%)	0.565
FUN	5	0 (0.0%)	5 (100.0%)	
HI	5	1 (20.0%)	4 (80.0%)	
Non neonatal	19	6 (31.6%)	13 (68.4%)	0.565
Neonatal	5	0 (0.0%)	5 (100.0%)	
HI	5	1 (20.0%)	4 (80.0%)	
Non TGFB	21	5 (23.8%)	16 (76.2%)	1.000
TGFB	3	1 (33.3%)	2 (66.7%)	
HI	5	1 (20.0%)	4 (80.0%)	

DN, dominant-negative; DN (-Cys), DN variants eliminating the disulfide-bridge forming cysteines; DN (CaB), DN variants affecting the conserved calcium-binding motif; DN (Others), DN variants affecting other residues; DN-CD, DN variants in tandem arrays of cb EGF-like domain; FUN-EGF, fibrillin unique N-terminal (FUN) and the first three epidermal growth factor (EGF)-like domains; HI, haplo-insufficiency; TGFB, TGF-β regulating.
