# Supplementary material for: Genotype Impacts Axial Length Growth in Pseudophakic Eyes of Marfan Syndrome
Source: Invest Ophthalmol Vis Sci. 2023 Jul 21;64(10):28. doi: 10.1167/iovs.64.10.28 (PMC10365134; doi:10.1167/iovs.64.10.28)
Supplement: Supplement 8 [file iovs-64-10-28_s008.pdf]

Supplementary Table S5. The difference between patients with severe and nonsevere AL elongation.

Variables	Mean ± standard deviation (range) or Median (IQR) or number (%)		*P* value
	Patients with nonsevere AL elongation (n = 22)	Patients with continued severe AL elongation (n = 8)	
Age/y	27.42 (18.25, 30.91)	22.50 (21.95, 27.67)	0.475
Sex			
Male	14 (77.8%)	4 (22.2%)	0.678
Female	8 (66.7%)	4 (33.3%)	
EL severity			
Mild	11 (84.6%)	2 (15.4%)	0.284
Moderate	6 (54.5%)	5 (45.5%)	
Severe	5 (83.3%)	1 (16.7%)	
Laterality			
Bilateral	20 (74.1%)	7 (25.9%)	1.000
Unilateral	2 (66.7%)	1 (33.3%)	
BCVA/LogMAR	0.40 (0.15, 0.52)	0.30 (0.22, 0.55)	1.000
SE/D	-9.72 ± 12.79	-9.73 ± 12.20	0.998
preAL/mm	26.24 ± 3.85	26.90 ± 3.13	0.667
preKm/D	41.25 ± 1.66	40.96 ± 0.91	0.639
pre corneal astigmatism/D	-1.60 (-2.53, -1.36)	-0.93 (-1.53, -0.55)	0.031
preACD/D	3.09 ± 0.30	3.38 ± 0.51	0.079
preLT/mm	4.04 (3.79, 4.43)	3.66 (2.12, 4.49)	0.210
preWTW/mm	11.99 ± 0.77	12.15 ± 0.52	0.653

ACD, anterior chamber depth; AL, axial length; BCVA, best-corrected visual acuity; D, diopter; EL, ectopia lentis; Km, median keratometry of meridian; LogMAR, logarithm

of the minimal angle of resolution; LT, lens thickness; IQR, interquartile range; SD, standard deviation; SE, spherical equivalent; WTW, white-to-white measurement.
